# Nucleolin Down-Regulation Is Involved in ADP-Induced Cell Cycle Arrest in S Phase and Cell Apoptosis in Vascular Endothelial Cells

**DOI:** 10.1371/journal.pone.0110101

**Published:** 2014-10-07

**Authors:** Wenmeng Wang, Junqing Luo, Fang Xiang, Xueting Liu, Manli Jiang, Lingjuan Liao, Jinyue Hu

**Affiliations:** 1 Department of Internal Medicine, Hunan Armed Police Force's Hospital, Changsha, Hunan, China; 2 Medical Research Center, Changsha Central Hospital, Changsha, Hunan, China; Ospedale Pediatrico Bambino Gesu', Italy

## Abstract

High concentration of extracellular ADP has been reported to induce cell apoptosis, but the molecular mechanisms remain not fully elucidated. In this study, we found by serendipity that ADP treatment of human umbilical vein endothelial cells (HUVEC) and human aortic endothelial cells (HAEC) down-regulated the protein level of nucleolin in a dose- and time-dependent manner. ADP treatment did not decrease the transcript level of nucloelin, suggesting that ADP might induce nucleolin protein degradation. HUVEC and HAEC expressed ADP receptor P2Y13 receptor, but did not express P2Y1 or P2Y12 receptors. However, P2Y1, 12, 13 receptor antagonists MRS2179, PSB0739, MRS2211 did not inhibit ADP-induced down-regulation of nucleolin. Moreover, MRS2211 itself down-regulated nucleolin protein level. In addition, 2-MeSADP, an agonist for P2Y1, 12 and 13 receptors, did not down-regulate nucleolin protein. These results suggested that ADP-induced nucleolin down-regulation was not due to the activation of P2Y1, 12, or 13 receptors. We also found that ADP treatment induced cell cycle arrest in S phase, cell apoptosis and cell proliferation inhibition via nucleolin down-regulation. The over-expression of nucleolin by gene transfer partly reversed ADP-induced cell cycle arrest, cell apoptosis and cell proliferation inhibition. Furthermore, ADP sensitized HUVEC to cisplatin-induced cell death by the down-regulation of Bcl-2 expression. Taken together, we found, for the first time to our knowledge, a novel mechanism by which ADP regulates cell proliferation by induction of cell cycle arrest and cell apoptosis via targeting nucelolin.

## Introduction

Nucleolin, an abundant, ubiquitously expressed protein, is composed of three main domains: a N-terminal segment with multiple phosphorylation sites, a central domain with four RNA-recognition motifs (RRMs) and a C-terminal arginine–glycine-rich (RGG) domain [1], [2]. Nucleolin is found in various cell compartments, especially in the nucleolus, of which it is a major component and functions as a prominent RNA-binding protein (RBP) to interacts with precursor ribosomal (r)RNA and is essential for rRNA biogenesis and rRNA transport to the cytoplasm [1], [3]. Accordingly, inactivation of nucleolin leads to nucleolar disruption, cell cycle arrest and defects in centrosome duplication [4]. Nucleolin was also found to function associated with binding DNA to induce chromatin decondensation by the remodelin complex SWI/SNF (switch/sucrose non-fermentable in yeast), facilitates transcription and modulates DNA replication [2], [5]. Recently, Nucleolin has been found on the cell surface, where it functions as a target for cancer therapy [6]–[11]. Nucleolin was also found to be related to viral infection [12], replication [13], [14], and to the efficient nuclear egress of viral nucleocapsids [15]. By binding mRNAs, nucleolin has been reported to regulate the expression of Bcl-2 and selenoprotein [16], [17].

Nucleotides are a class of ubiquitous and potent extracellular signaling molecules for the regulation of cell proliferation, cell differentiation, cell chemotaxis, cytokine production and reactive oxygen generation [18], [19] through a specific class of plasma membrane receptors called purinergic P2 receptors, which are subdivided into two distinct categories, the metabotropic G protein-coupled (P2Y) receptors and the ionotropic ligand-gated channel (P2X) receptors [18]–[20]. Adenosine diphosphate (ADP) can be released from platelets following endothelial cell damage, in response to all stimulatory platelet agonists, and acts as a secondary positive feedback mediator of platelet activation [21] through two G protein-coupled receptors, the Gq-coupled P2Y1 receptor activates phospholipase C isoforms leading to formation of the second messengers 1,2-diacylglycerol and inositol 1,4,5-trisphosphate, which activate protein kinase C (PKC) and mobilize Ca^2+^, respectively, and the Gi-coupled P2Y12 receptor inhibits adenylyl cyclase and activates PI3-kinase [22], [23]. Recently, ADP had been reported to mediate inhibition of insulin secretion, to regulate the endocytosis of hepatic high density lipoprotein through the Gi/o-coupled P2Y13 receptor [24], [25]. In addition, ADP functions to regulate cell proliferation [26]–[30], cell apoptosis [31]–[34], cell migration [35]–[37], the generation of thromboxane A2 [21], the ATP release from human red blood cells [38], and the antigen endocytosis in dendritic cells [39]. However, the effect of ADP on cell proliferation is contradictory, and the molecular mechanisms are not fully elucidated.

In the current study, we found that ADP down-regulated the protein level of nucleolin in a P2Y1, 12, and 13 receptor-independent manner. Nucleolin down-regulation was involved in ADP-induced cell cycle arrest, cell apoptosis and finally cell proliferation inhibition. Furthermore, ADP-induced down-regulation of nucleolin sensitized HUVEC to cisplatin-induced cell death.

## Materials and Methods

### Reagents and antibodies

ADP, ATP, UDP, and UTP were purchased from Sigma-Aldrich (St. Louis, MO). Rabbit anti-human Bcl-2, total ERK, phospho-ERK antibodies, Rabbit anti-human nucleolin antibody, and ERK inhibitor U0126 were purchased from Cell Signaling Technology (Beverly, MA). P2Y1, 12, 13 agonist 2-MeSADP, P2Y1 selective inhibitor MRS2179, P2Y12 potential inhibitor PSB0739, P2Y13 competitive inhibitor MRS2211 were purchased from Tocris (Bristol, UK). Mammalian expression plasmid pReceive-M29 coding for eGFP-nucleolin fusion protein was purchased from GeneCopoeia (Germantown, MD).

### Cell culture

Primary human aortic endothelial cells (HAEC, ScienCell) were plated on culture dishes pre-coated with 10 ng/ml fibronectin (Millipore) and cultured in endothelial cell medium (ECM, ScienCell) supplemented with 5% fetal calf serum (FCS), 1% endothelial cell growth supplement (ECGS), 100 units/ml penicillin, and 100 µg/ml streptomycin [40]. Cells were used from passages 3 to 6 in all experiments. Immortalized human umbilical vein endothelial cells (HUVEC), monocyte cell line THP1, and cervical cancer cell line Caski were purchased from ATCC (Manassas, VA) and cultured in DMEM (HUVEC, Caski), or RPMI 1640 (THP1) containing 10% FCS and antibiotics. All cells were cultured in a humidified atmosphere with 5% CO_2_ at 37°C.

### CCK-8 cell proliferation assay

Cell proliferation was determined using a cell counting kit-8 (CCK-8) (Dojido, Kumamoto, Japan) assay according to the manufacturer's instructions. Briefly, 2,000–5,000 cells in 100 µl of medium were plated on a 96-well plate and cultured for 1–6 days. After the incubation period, 10 µl of CCK-8 was added to each well, and cells were further incubated for 1 h at 37°C. Absorbance was then measured at 450 nm using a microplate reader (PerkinElmer, USA).

### Flow cytometric analysis

Cell death was detected by fluorescein isothiocyanate (FITC)-annexin V/propidium iodide (PI) staining. Briefly, 1−2×10^6^ cells were washed twice with PBS, then labeled with FITC-annexin V and PI in binding buffer according to manufacturer's instructions. Fluorescence signals were detected on a FACScan (BD Bioscience, San Jose, CA). The log of FITC-annexin V–fluorescence was displayed on the x-axis, and the log of PI fluorescence was displayed on the y-axis. For each analysis, 10,000 events were recorded.

Cell cycle status was analyzed using propidium iodide (PI) staining. Briefly, Cells were cultured in serum-free medium for 24 h for synchronization. Then 1−2×10^5^ cells were plated in 6-well plates and incubated with or without various concentrations of nucleotides. After two washes with ice-cold PBS, the adhered cells were collected and fixed in ethanol overnight at 4°C and incubated with a mixture of 50 µg/ml PI (Sigma-Aldrich) and 25 µg/ml RNase A (Sigma-Aldrich) at 37°C for 30 min. The level of PI fluorescence was measured with a FACScan, and the proportion of cells in Go/G_1_, S, and G_2_/M phases was measured.

### RT-PCR

Total RNA was extracted from 1−5×10^6^ cells using Trizol (Life Technologies, Gaithersburg, MD) according to the manufacturer's instructions. mRNA was reverse transcribed with RevertAid (MBI Fermemtas, Burlington Ontario, Canada) at 42°C for 60 min, and the resulting cDNA was subjected to PCR (94°C for 1 min followed by 20–40 cycles at 94°C for 30 sec, 60°C for 30 sec, 68°C for 90 sec and an extension cycle for 10 min at 68°C). PCR products were separated on 1.0% agarose gels and visualized with ethidium bromide. Forward and reverse primer pairs are listed (5′ to 3′) as follows:

β-actin-F: ATTGCCGACAGGATGCAGAAG

β-actin-R: CCATGCCAATCTCATCTTGT

Bcl-2-F: CGTTTGGCAGTGCAATGGT

Bcl-2-R: TTCTTGATTGAGCGAGCCTT

GAPDH-F: AGAAGGCTGGGGCTCATTT

GAPDHA-R: CCATCACGCCACAGTTTCC

P2Y1-F: ATGTGTGCTTTCAATGACAGGGTTT

P2Y1-R: TGTGGATGTGGGCATTTCTACTTCT

P2Y12-F: TGTTGTCATCTGGGCATTCA

P2Y12-R: TTACCTACACCCCTCGTTCTT

P2Y13-F: GGTGTTTGTTCACATCCCCAG

P2Y13-R: CTTTAAGGAAGCACACTTTTTCAC

### Western blot

1−2×10^6^ cells were lysed in 200 µl lysis buffer (20 mM Tris, pH 7.5, 150 mM NaCl, 1% Triton X-100, 1 mM EDTA, 1 mM sodium pyrophosphate, 1 mM β-glycerophosphate, 1 mM Na3VO4, 1 µg/ml leupeptin). The cell lysate was centrifuged at 12,000×g at 4°C for 5 min. Equivalent amounts of protein were electrophoresed on 10% SDS-PAGE gels and transferred onto Immobilon P membranes (Millipore). The membranes were blocked by incubation with 3% nonfat dry milk for 1 h at room temperature and then incubated with primary antibodies (1∶200–1000) in PBS containing 0.01% Tween 20 overnight at 4°C. After incubation with a horseradish peroxidase–conjugated secondary antibody (1∶2000), the protein bands were detected with SuperSignal Chemiluminescent Substrate Stable Peroxide Solution (Pierce) and BIOMAX-MR film (Eastman Kodak). When necessary, the membranes were stripped with Restore Western Blot Stripping Buffer (Pierce) and re-probed with antibodies against various cellular proteins.

### Plasmid transfection

Cells cultured in six-well plates were transfected with 1 µg of the plasmid containing sequence coding for the GFP-Nucleolin fusion protein using Lipofectamine 2000 (Invitrogen) according to the manufacturer's instructions. Expression of GFP-nuclolin in the transfected cells was examined by fluorescent microscope 48 h after transfection. For stable transfection, G418-resistant cells were selected after incubation with 800 µg/ml G418 for 3 weeks.

### Quantitative Real Time RT-PCR (qRT-PCR)

The qRT-PCR was performed as described by Sun *et al* [41]. Briefly, total RNA was isolated and reverse transcribed as above. The cDNA was amplified using TaqMan Universal PCR master mix (Applied Biosystems, Foster City, CA, USA) and an ABI Prism 7500 sequence detection system (Applied Biosystems). Amplification of the target genes was normalized using the amplification levels of glyceraldehyde-3-phosphate dehydrogenase (*GAPDH*) as an endogenous control. The efficiency of the PCR was tested by amplification of the target from serially diluted cDNA generated from the reverse transcription of a stock set of human RNA. Data analysis and calculations were performed using the 2^−ΔΔ*CT*^ comparative method, as described by the manufacturer. Gene expression is shown as the fold induction of a gene measured in ADP-treated samples, relative to samples cultured with medium. The same primer pairs were used as described in RT-PCR.

### Scratch wound–healing assay

The scratch wound-healing assay was performed as described by Song *et al*. [42]. Briefly, cells, cultured overnight, were allowed to reach 100% confluence. A 20- µl pipette tip was used to scratch and create a wound in the confluent monolayer. Detached cells were immediately removed by replacement of medium. Cells were then treated with 1–100 µM ADP for 24 h. Wound repair images were captured at time point 0, 6, 12, and 24 h, and the repair percentages were calculated. All experiments were performed in triplicate.

### Statistical analysis

All experiments were performed at least three times, and the representative results were shown. Results are expressed as the mean plus or minus the standard deviation (SD). Differences between two groups were examined for statistical significance using Student's *t* test, and *p* values equal to or less than 0.05 were considered statistically significant.(n = 3 for each qRT-PCR test).

## Results

### ADP down-regulates protein level of nucleolin

**Figure 1:**
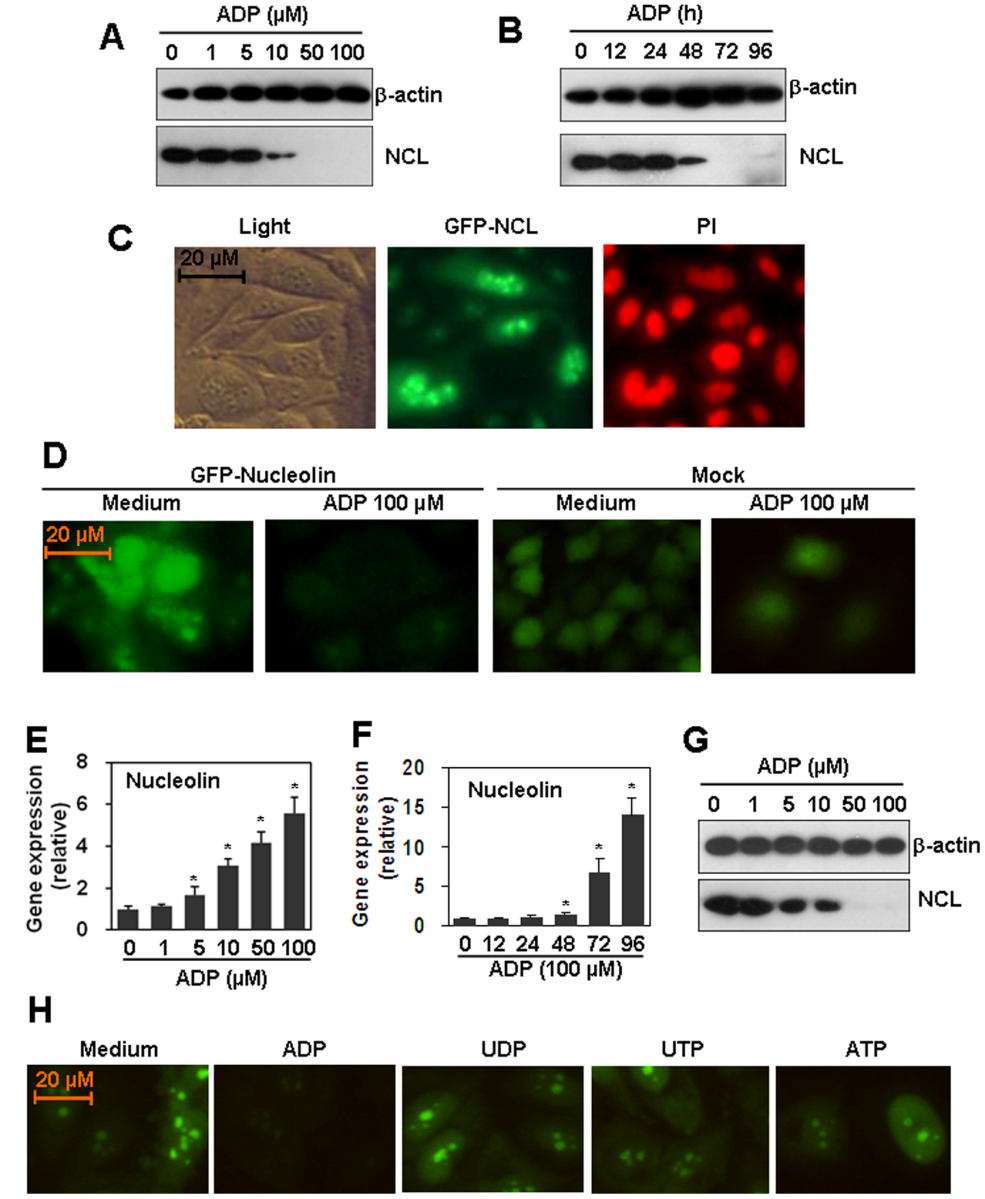
ADP down-regulates nucleolin protein level. (A) Dose-response. HUVEC were treated with the indicated concentrations of ADP for 72 h. Medium was replaced with fresh medium containing ADP every day. The nucleolin protein was detected by western blot. β-actin protein was detected as a loading control. (B) Time course. HUVEC were treated with 100 µM ADP for the indicated time periods. Medium was replaced with fresh medium containing ADP every day. The nucleolin protein was detected by western blot. β-actin protein was detected as a loading control. (C) Intracellular location of nucleolin. HUVEC were transfected with GFP-nucleolin-expression plasmid. G418-resistant cells were selected and GFP-nucleolin was detected by fluorescence microscopy. Cells were counterstained with 50 µg/ml Propidium Iodide (PI) to show nucleus staining. (D) ADP down-regulated over-expressed nucleolin. Nucleolin-over-expressed HUVEC were treated with 100 µM ADP for 72 h. Medium was replaced with fresh medium containing ADP every day. The GFP-nucleolin was detected by fluorescence microscope. Cells, mock-transfected with GFP-expression plasmid, were treated as control. (E) The effect of ADP on nucleolin mRNA levels in HUVEC. Cells were treated with the indicated concentrations of ADP for 72 h. Medium was replaced with fresh medium containing ADP every day. The mRNA level of nucleolin was detected by qRT-PCR. * *P*<0.05 compared with the control group. (F) The effect of ADP on nucleolin mRNA level in HAEC. Cells were treated with 100 µM ADP for the indicated time periods. Medium was replaced with fresh medium containing ADP every day. The mRNA level of nucleolin was detected by qRT-PCR. * *P*<0.05 compared with the control group. (G) The effect of ADP on nucleolin protein level primary human aortic endothelial cells (HAEC). (H) The effect of ADP, UDP, UTP, and ATP on nucleolin expression. Nucleolin-over-expressed HUVEC were treated with 100 µM ADP, or UDP, or UTP, or ATP for 72 h. Medium was replaced with fresh medium containing nucleotide every day. The GFP-nucleolin was detected by fluorescence microscope.

By serendipity, we found that ADP treatment of HUVEC down-regulated the protein level of nucleolin. As shown in Figures 1A–B, ADP decreased nucleolin level in a dose- and time-dependent manner. The ADP concentrations for nucleolin down-regulation were from 10 to 100 µM (Figure 1A), and the treatment time for nucleolin down-regulation was from 48 to 96 h (Figure 1B). Then HUVEC were transfected with GFP-nucleolin-expression plasmid to detect the nucleolin distribution. The results from fluorescence microscope showed that HUVEC expressed nuclolin in both nucleus and cytoplasm (Figure 1C). Fluorescence microscope results showed that ADP treatment also down-regulated the GFP-nucleolin level in nucleolin-overexpressed HUVEC cells (Figure 1D). As control, when cells were mock-transfected with GFP-expression plasmid, the cell number was decreased, but the GFP level was not regulated by the treatment with ADP (Figure 1D). To test whether ADP down-regulated nucleolin expression via inhibition of nucleolin transcription, HUVEC were treated with various concentrations of ADP for 72 h, and mRNA levels of nucleolin were detected by qRT-PCR. Unexpectedly, ADP did not down-regulate, but up-regulated nucelolin transcript levels (Figure 1E) in a dose-dependent manner. ADP-induced increase of nucleolin transcript was also time-dependent. 100 µM ADP up-regulated nucleolin mRNA levels significantly from 48 to 96 h (Figure 1F). These results suggested that ADP induced a post-transcriptional down-regulation of nucleolin protein level. In addition, we also found that ADP down-regulated nucleolin protein level in primary human aortic endothelial cells (Figure 1G). Meanwhile, we detected the effect of UDP, UTP, and ATP on the nucleolin expression in nucleolin over-expressed HUVEC. The results showed that UDP, or UTP, or ATP did not regulated the expression of nucleolin (Figure 1H).

### P2Y1, 12, and 13 receptors are not responsible for ADP-induced down-regulation of nucleolin protein

**Figure 2:**
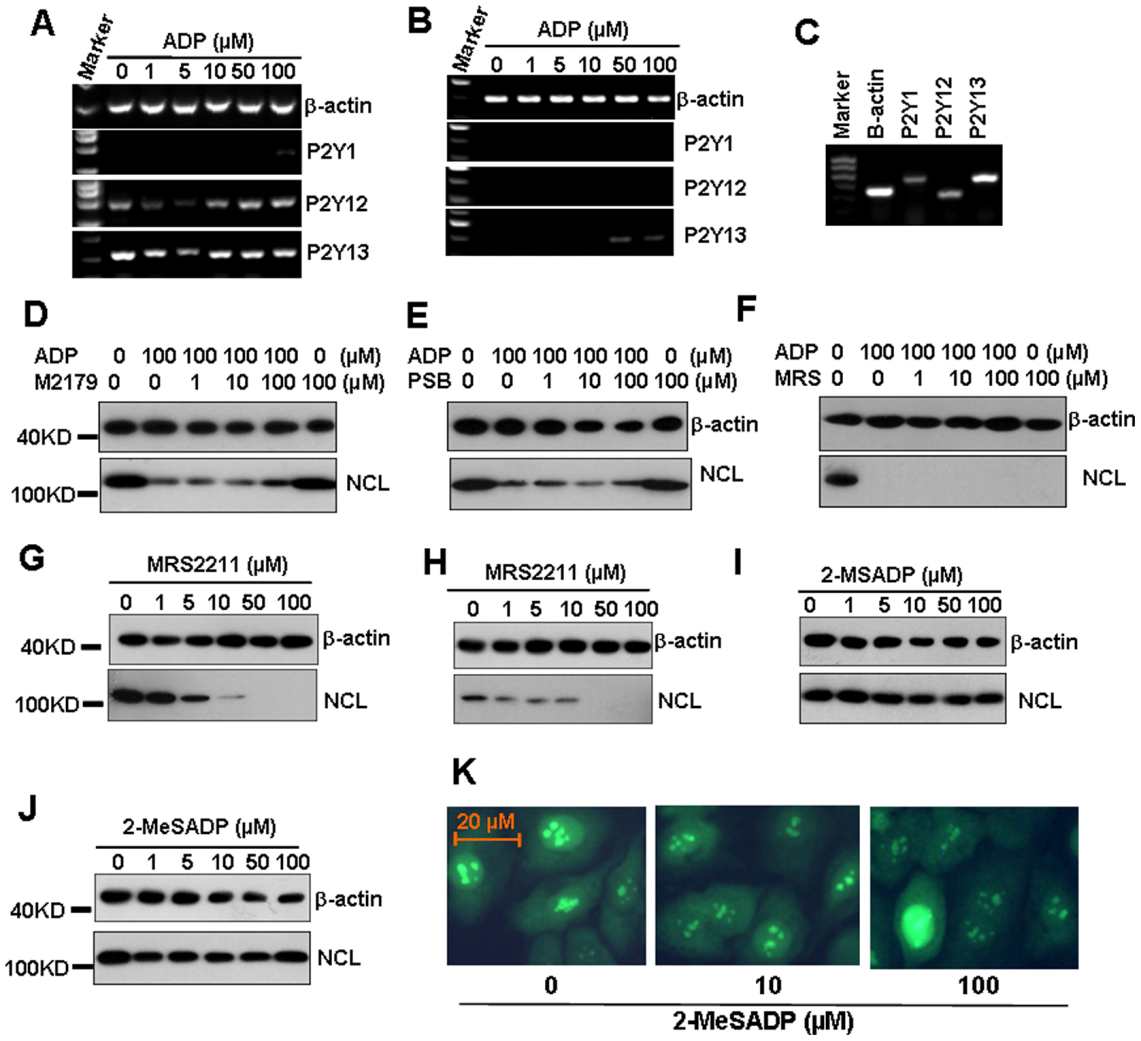
P2Y1, 12, and 13 receptors are not involved in ADP-induced nucleolin down-regulation. (A, B) The mRNA levels of P2Y1, 12, and 13 receptors in HUVEC (A) and HAEC (B). Cells were treated with the indicated concentrations of ADP for 24 h. The mRNA levels of P2Y1, 12 and 13 receptors were detected by RT-PCR. β-actin transcript was detected as a loading control. (C) The mRNA levels of P2Y1, P2Y12, and P2Y13 receptors in 5–8F cells. (D) The effect of P2Y1 receptor inhibitor MRS2179 (M2179) on ADP-induced down-regulation of nucleolin protein. HUVEC, pre-treated with the indicated concentrations of MRS2179 for 1 h, were re-treated with 100 µM ADP for 72 h. The nucleolin protein levels were detected by western blot. β-actin transcript was detected as a loading control. (E) The effect of P2Y12 receptor inhibitor PSB0739 (PSB) on ADP-induced down-regulation of nucleolin protein. (F) The effect of P2Y13 receptor antagonist MRS2211 (MRS) on ADP-induced down-regulation of nucleolin. (G, H) The effect of MRS2211 on the nucleolin protein levels in HUVEC (G) and HAEC (H). (I, J) The effect of P2Y13 receptor agonist 2-MeSADP on nucleolin protein levels in HUVEC (I) and HAEC (J). (K) 2-MeSADP did not regulate over-expressed nucleolin protein. Nucleolin-over-expressed HUVEC were treated with the indicated concentrations of ADP for 72 h. The GFP-nucleolin expression was detected by fluorescence microscope.

**Figure 3:**
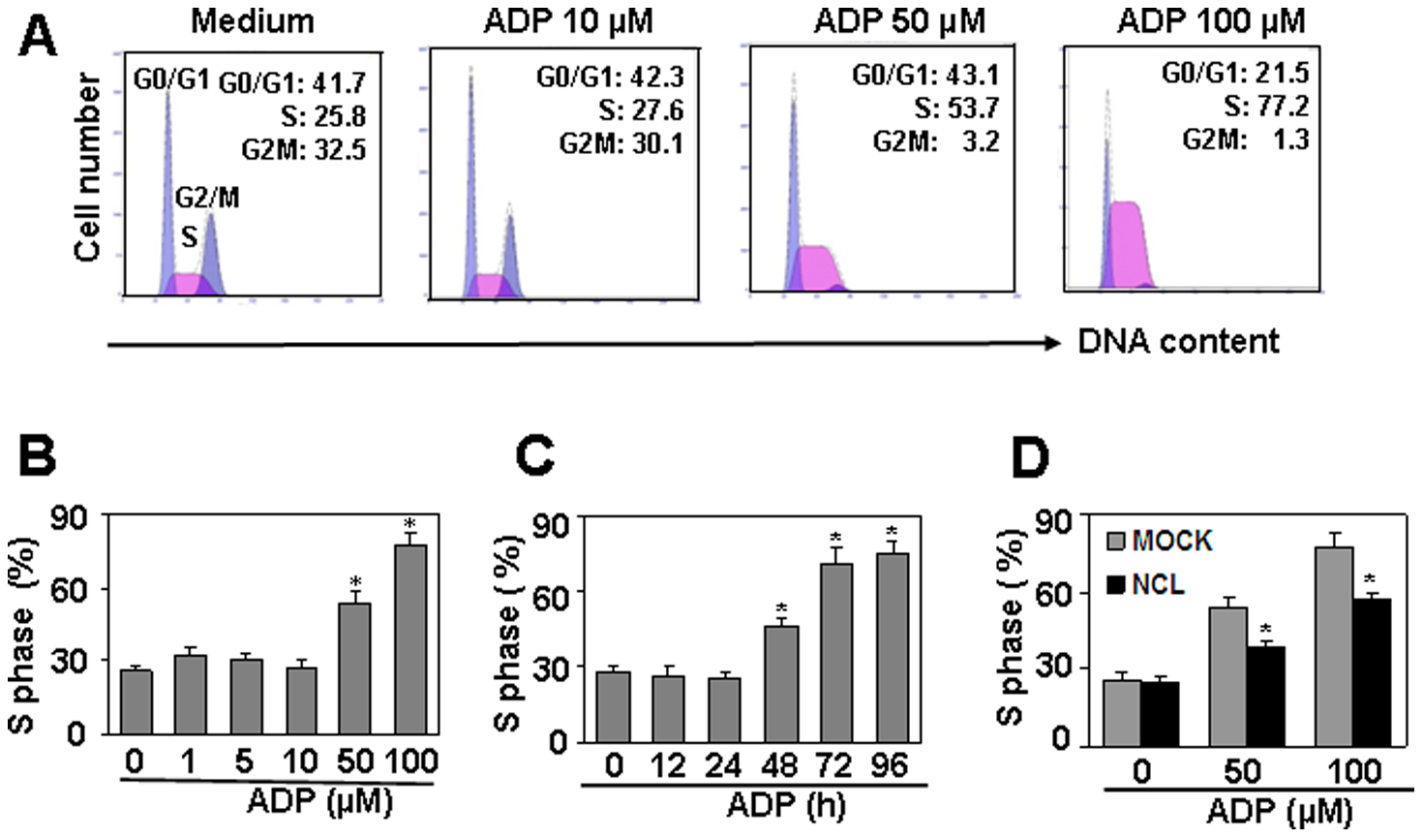
Nucleolin protein down-regulation is involved in ADP-induced cell cycle arrest in S phase. (A) ADP induced cell cycle arrest in S phase. HUVEC were treated with the indicated concentrations of ADP for 72 h. Medium was replaced with fresh medium containing ADP every day. The detached cells were removed, and the adhered cells were collected for cell cycle test by PI staining. (B) Quantitation of cell cycle arrest in S pahse induced by ADP in (A). * *P*<0.05 compared with the control group. (C) ADP induced time-dependent cell arrest in S phase in HUVEC. * *P*<0.05 compared with the control group. (D) Nucleolin over-expression partly reversed ADP-induced cell cycle arrest in S phase. Nucleolin-over-expressed HUVEC were treated with the indicated concentrations of ADP for 72 h. Medium was replaced with fresh medium containing ADP every day. The cell cycles were measured by PI staining. Mock-transfected HUVEC were used as controls. * *P*<0.05 compared with the control groups.

P2Y1, 12, and 13 receptors are ADP-preferring P2Y receptors [18]. To prove whether these receptors are responsible for ADP-induced down-regulation of nucleolin protein, we first detected the mRNA levels of P2Y1, 12, and 13 receptors in endothelial cells. The RT-PCR results showed that HUVEC expressed very low level of P2Y1 receptor, moderate level of P2Y12 receptor, and high level of P2Y13 receptor (Figure 2A). HAEC expressed low level of P2Y13 receptor, but did not express P2Y1, or P2Y12 receptors (Figure 2B). As control, nasopharygeal carcinoma 5–8F cells [42]–[43] expressed all P2Y1, 12, and 13 receptors (Figure 3C). Then HUVEC cells were pre-treated with P2Y1 receptor inhibitor MRS2179, P2Y12 receptor inhibitor PSB0739, P2Y13 receptor inhibitor MRS2211 respectively, for 30 min to test the effect of receptor inhibition on ADP-induced nucleolin down-regulation. Unexpectedly, the pre-treatment of HUVEC with every inhibitor did not inhibit ADP-induced down-regulation of nucleolin (Figure 2D–F). Moreover, MRS2211 itself induced the down-regulation of nucleolin in HUVEC (Figure 2G) and HAEC (Figure 2H). Then, we tested the effect of 2-MeSADP, another agonist for P2Y1, 12 and 13 receptors, on the expression of nucleolin. The results showed that the treatment of both HUVEC (Figure 2I) and HAEC (Figure 2J) with various concentrations of 2-MeSADP for 72 h did not impact nucleolin protein levels. Meanwhile, 2-MeSADP did not regulate the over-expression nucleolin in GFP-nucleolin-transfected HUVEC (Figure 2K). These results suggested that P2Y1, 12, and 13 receptors are not involved in ADP-induced down-regulation of nucleolin protein.

### Nucleolin is involved in ADP-induced cell cycle arrest in S phase

By targeting nucleolin protein, G-rich oligonucleotides (GROs) has been reported to induce S phase cell cycle arrest [44]–[45]. As ADP down-regulated nucleolin protein level significantly, we supposed that ADP may induce cell cycle arrest as GROs did. This hypothesis was proved by the observation that the treatment of HUVEC with ADP for 72 h induced cell cycle arrest in S phase (Figure 3A). The cell proportion in S phase was increased from 25.8% to 77.2%, and the increase was dose- and time-dependent (Figures 3B–C). To test whether nucleolin down-regulation was involved in the induction of cell cycle arrest in S phase, nucleolin-over-expressed HUVEC was treated with ADP, and the cell proportion in each cell cycle was detected by PI staining. The results showed that nucleolin over-expression partly reversed the induction of cell cycle arrest in S phase (Figure 3D). These results suggested that ADP induced cell cycle arrest in S phase via targeting nucleolin.

### Nucleolin is involved in ADP-induced cell apoptosis

**Figure 4:**
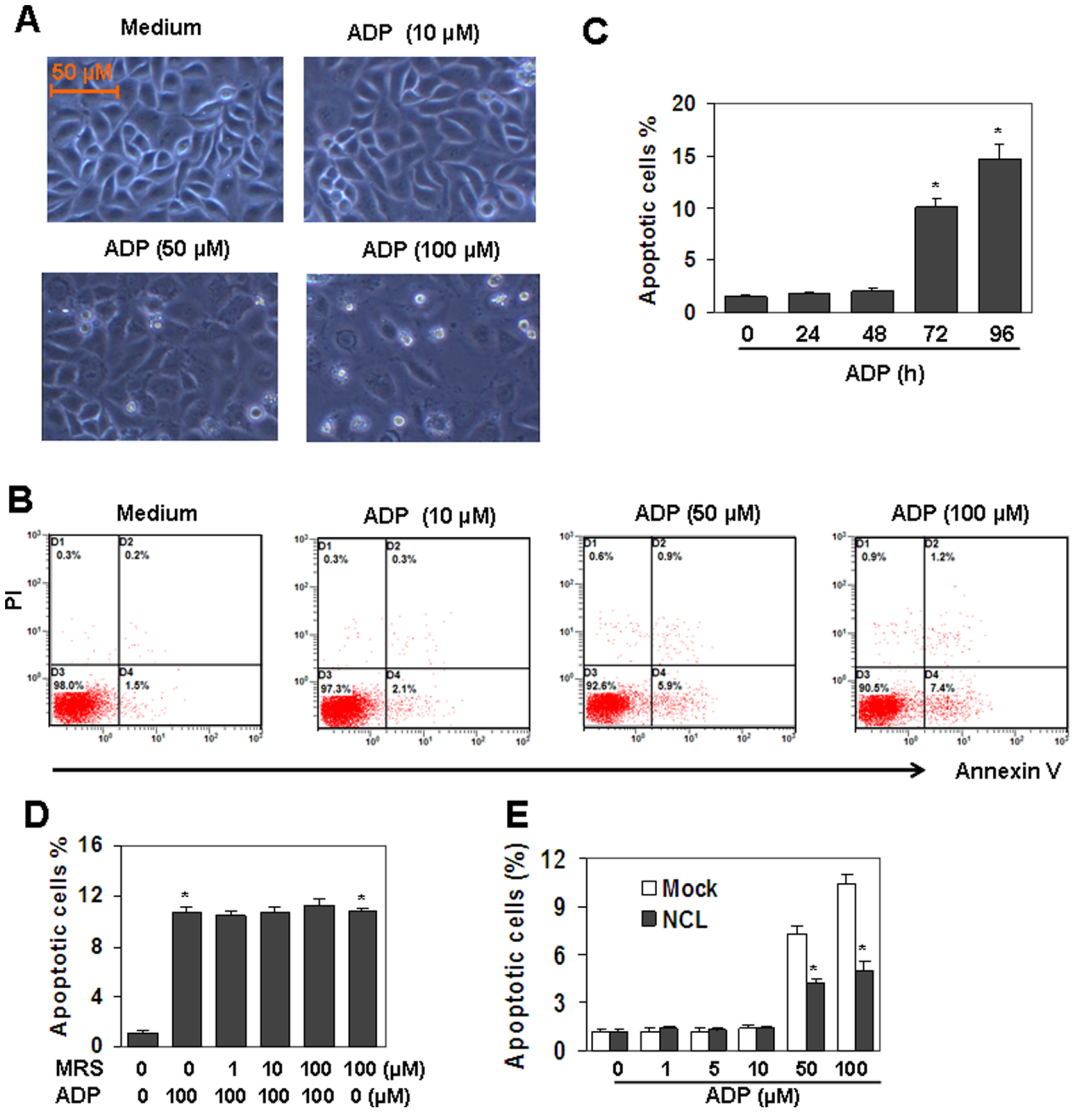
Nucleolin protein down-regulation is involved in ADP-induced cell apoptosis. (A) ADP induced cell detachment in HUVEC. Cells were treated with indicated concentrations of ADP for 72 h. Medium was replaced with fresh medium containing ADP every day. The cells in representative fields were photographed. (B) ADP induced apoptosis in HUVEC. Cells were treated as same as (A). Unfixed cells were stained with FITC-annexin V/PI. Cell apoptosis was measured by flow cytometry analysis. (C) ADP induced time-dependent cell apoptosis. HUVEC were cultured with 100 µM ADP for indicated time periods. Medium was replaced with fresh medium containing ADP every day. Cell apoptosis was measured as same as (B). * *P*<0.05 compared with the control group. (D) MRS2211 (MRS) did not reverse ADP-induced cell death. HUVEC, pre-treated with the indicated concentrations of MRS2211 for 30 min, were re-treated with 100 µM ADP for 72 h. Cell death was measured by FITC-annexin V/PI staining. * *P*<0.05 compared with the control group. (E) Nuleolin-over-expression reversed ADP-induced cell death. Nucleolin-over-expressed HUVEC were treated with the indicated concentration of ADP for 72 h. Medium was replaced with fresh medium containing ADP every day. Cell death was measured by FITC-annexin V/PI staining. Mock-transfected HUVEC were used as control. * *P*<0.05 compared with the control groups.

Nucleolin has been reported to be related to the induction of cell apoptosis [46], [47]. The down-regulation of nucleolin induced by ADP prompted us to detect the effect of ADP on cell apoptosis. We found that the treatment of HUVEC with 10–100 µM ADP did not induce cell morphological changes at 24 h and 48 h, but from 72 h, a fraction of cells underwent detachment (Figure 4A). To quantify the fraction of dead cells, we stained unfixed cells treated by ADP for 72 h with FITC-annexin V/PI and performed flow cytometry analysis. Figure 4B showed the FITC-annexin V/PI profiles of HUVEC in response to the increasing concentrations of ADP. About 10% of endothelial cells underwent cell apoptosis by the treatment of ADP for 72 h. Then, we treated HUVEC with 100 µM ADP for 24 to 96 h, and the results showed that 100 µM ADP did not induce cell apoptosis at 24 h and 48 h, but from 72 h to 96 h, apoptosis was induced up to 10% of cells (Figure 4C). To test the involvement of P2Y13 receptor in ADP-induced cell death, HUVEC, pre-treated with 0.1–10 µM P2Y13 receptor antagonist MRS2211 for 30 min, were then re-stimulated with 100 µM ADP, and the cell apoptosis was measured by FACS. The results showed that MRS2211 pre-treatment did not decrease ADP-induced cell death, suggesting that P2Y13 receptor was not involved in ADP-induced cell death (Figure 4D). To test the involvement of nucleolin in ADP-induced cell death, nucleolin over-expressed HUVEC were treated with 1–100 µM of ADP for 72 h. FACS results showed that cell death in nucleolin over-expressed cells was down-regulated significantly than that in mock-transfected cells (Figure 4E). These results suggested that nucleolin down-regulation was involved in ADP-induced cell death.

### Nucleolin is involved in ADP-induced cell proliferation inhibition

**Figure 5:**
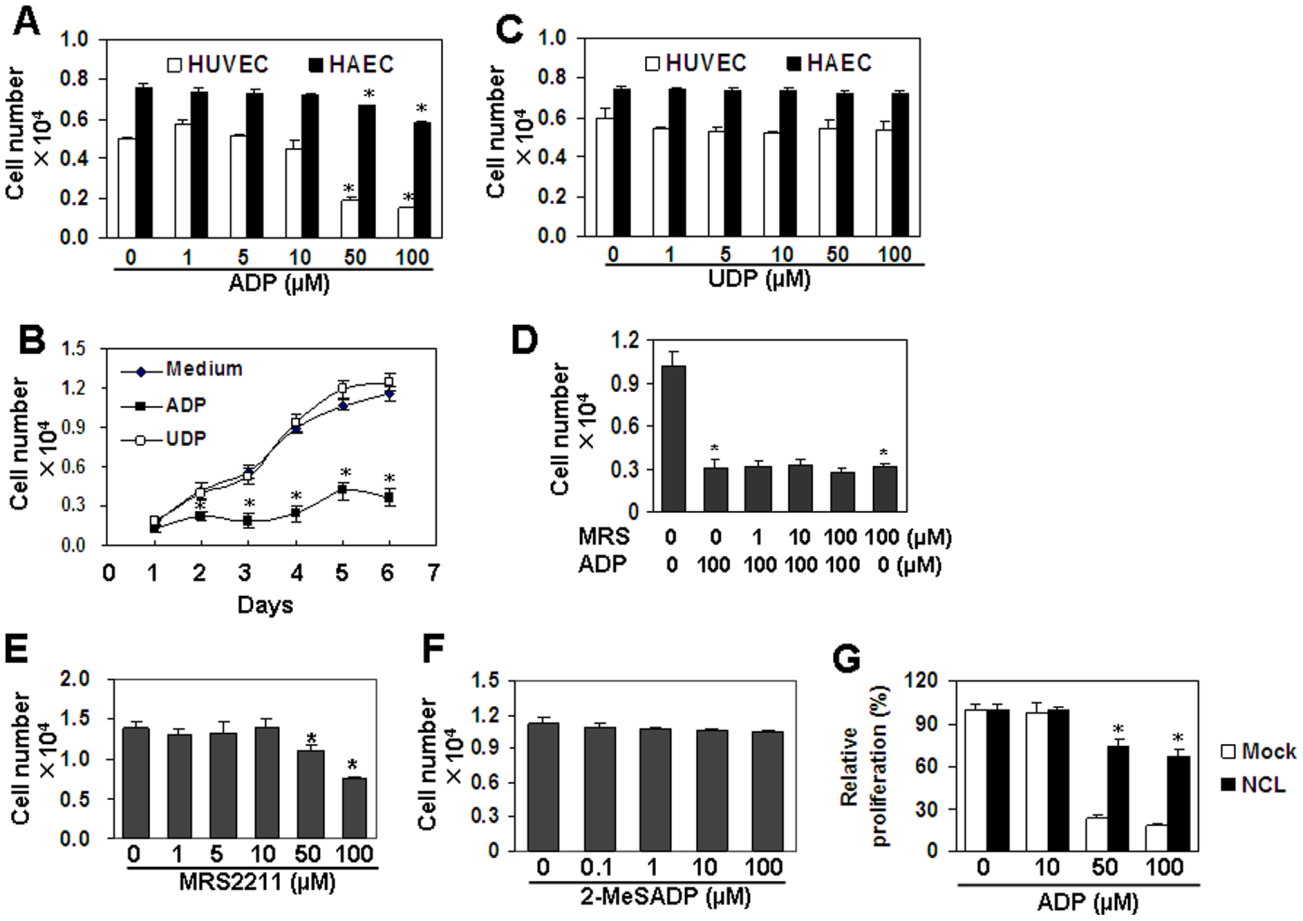
Nucleolin protein down-regulation is involved in ADP-induced inhibition of cell proliferation. (A) Endothelial cell proliferation in response to ADP. HUVEC and HAEC were treated with the indicated concentrations of ADP for 72 h. Medium was replaced with fresh medium containing ADP every day. Cell number was measured with CCK-8 assay. * *P*<0.05 compared with the control groups. (B) The effect of ADP and UDP on HUVEC proliferation. HUVEC were treated with 100 µM ADP or UDP for the indicated time periods. Medium was replaced with fresh medium containing ADP or UDP every day. Cell number was measured with CCK-8 assay. * *P*<0.05 compared with the control groups. (C) Endothelial cell proliferation in response to UDP. * *P*<0.05 compared with the control groups. (D) MRS2211 (MRS) did not reverse ADP-induced inhibition of cell proliferation. HUVEC, pre-treated with the indicated concentrations of MRS2211 for 30 min, were re-treated with ADP for 72 h. Medium was replaced with fresh medium containing MRS2211 and ADP every day. Cell number was measured with CCK-8 assay. * *P*<0.05 compared with the control groups. (E) MRS2211 (MRS) induced inhibition of cell proliferation. HUVEC were treated with the indicated concentrations of MRS2211 for 72 h. Medium was replaced with fresh medium containing MRS2211 every day. Cell number was measured with CCK-8 assay. * *P*<0.05 compared with the control group. (F) 2-MeSADP did not induce cell proliferation inhibition. HUVEC were treated with the indicated concentrations of 2-MeSADP for 72 h. Medium was replaced with fresh medium with 2-MeSADP every day. Cell numbers were measured by CCK-8 assay. (G) Nucelolin-over-expression partly reversed ADP-induced inhibition of cell proliferation. Nucleolin-over-expressed HUVEC were treated with the indicated concentrations of nucleolin for 72 h. Medium was replaced with fresh medium containing ADP every day. Mock-treansfected HUVEC were used as control. Cell number was measured with CCK-8 assay. * *P*<0.05 compared with the control groups.

Cell cycle arrest and cell apoptosis are mechanisms to regulate cell proliferation. We detected the effect of ADP on HUVEC and HAEC proliferation by CCK-8 assay. We found that ADP induced dose- and time-dependent inhibition of cell proliferation (Figures 5A–B). As a control, UDP did not induce inhibition of cell proliferation (Figure 5C). In addition, MRS2211 pre-treatment of HUVEC did not reverse ADP-induced inhibition of cell proliferation (Figure 5D). Moreover, MRS2211 itself induced cell proliferation inhibition at 50–100 µM (Figure 5D–E). And 2-MeSADP, an agonist for P2Y1, 12, 13 receptors, did not induce cell proliferation inhibition (Figure 5F). To test whether nucleolin is involved in the inhibition of cell proliferation, nucleolin over-expressed HUVEC were treated with 10–100 µM of ADP for 72 h, and cell proliferation was measured by CCK-8. The results showed that the inhibition of cell proliferation was reversed significantly in nucleolin-over-expressed HUVEC (Figure 5G). These results suggested that ADP-induced cell proliferation inhibition is nucleolin-dependent, but P2Y1, 12, and 13 receptors-independent.

### The effect of ERK signaling blocking on ADP-induced cell proliferation inhibition

**Figure 6:**
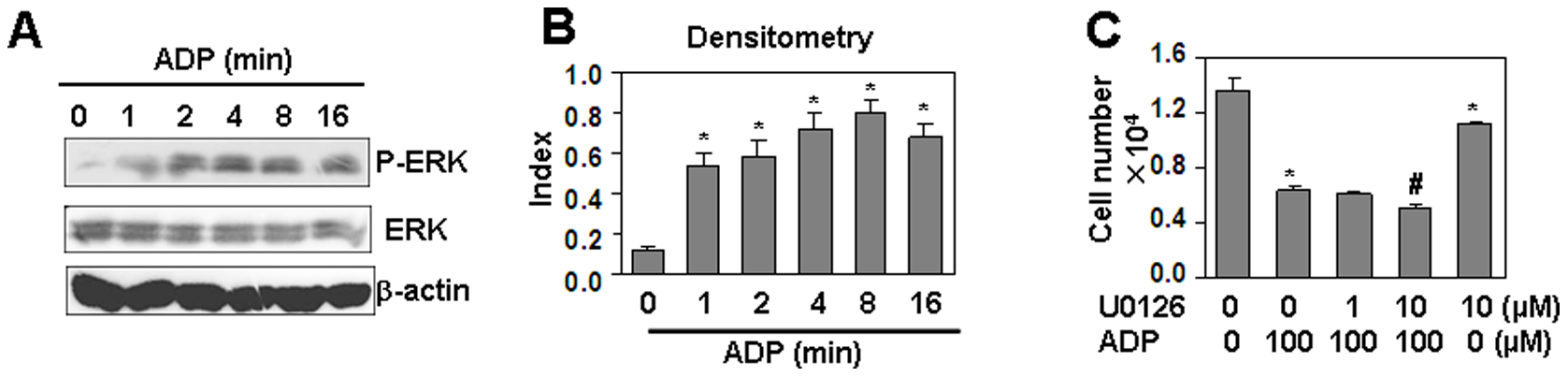
ERK pathway is not involved in ADP-induced cell proliferation inhibition. (A) ADP induced phosphorylation of ERK. HUVEC, starved overnight with serum-free medium, were treated with 100 µM ADP, and lysed at the indicated time points. Western blot was performed for the detection of phospho-ERK1/2 and total ERK1/2 respectively. β-actin protein was detected as a loading control. (B) Quantitation of phosphorylated ERK normalized to total ERK in (A). * *P*<0.05 compared with the control group. (C) The effect of ERK inhibitor, U0126, on ADP-induced cell proliferation inhibition. HUVEC, pre-treated with the indicated concentrations of U0126 for 30 min, were re-treated with the indicated concentrations of ADP for 24 h, followed with the same treatment once a day in the next 2 days. Cell number was measured by CCK-8 assay. * *P*<0.05 compared with the non-treated group. # *P*<0.05 compared with ADP-treated alone group.

ADP has been reported to activate ERK1/2 signal pathway [48]–[49] by binding its P2Y receptors. We tested the effect of ADP on ERK activation in HUVEC. The results showed that ADP induced ERK1/2 phosphorylation in a time-dependent manner in HUVEC (Figures 6A–B). However, when HUVEC, pre-treated with ERK1/2 inhibitor, U0126, were re-treated with ADP, the cell proliferation inhibition induced by ADP was not reversed (Figure 6C). These results suggested that ERK pathway did not contributed to ADP-induced cell proliferation inhibition.

### ADP down-regulates Bcl-2 expression and sensitizes HUVEC to cisplatin-induced cell death

**Figure 7:**
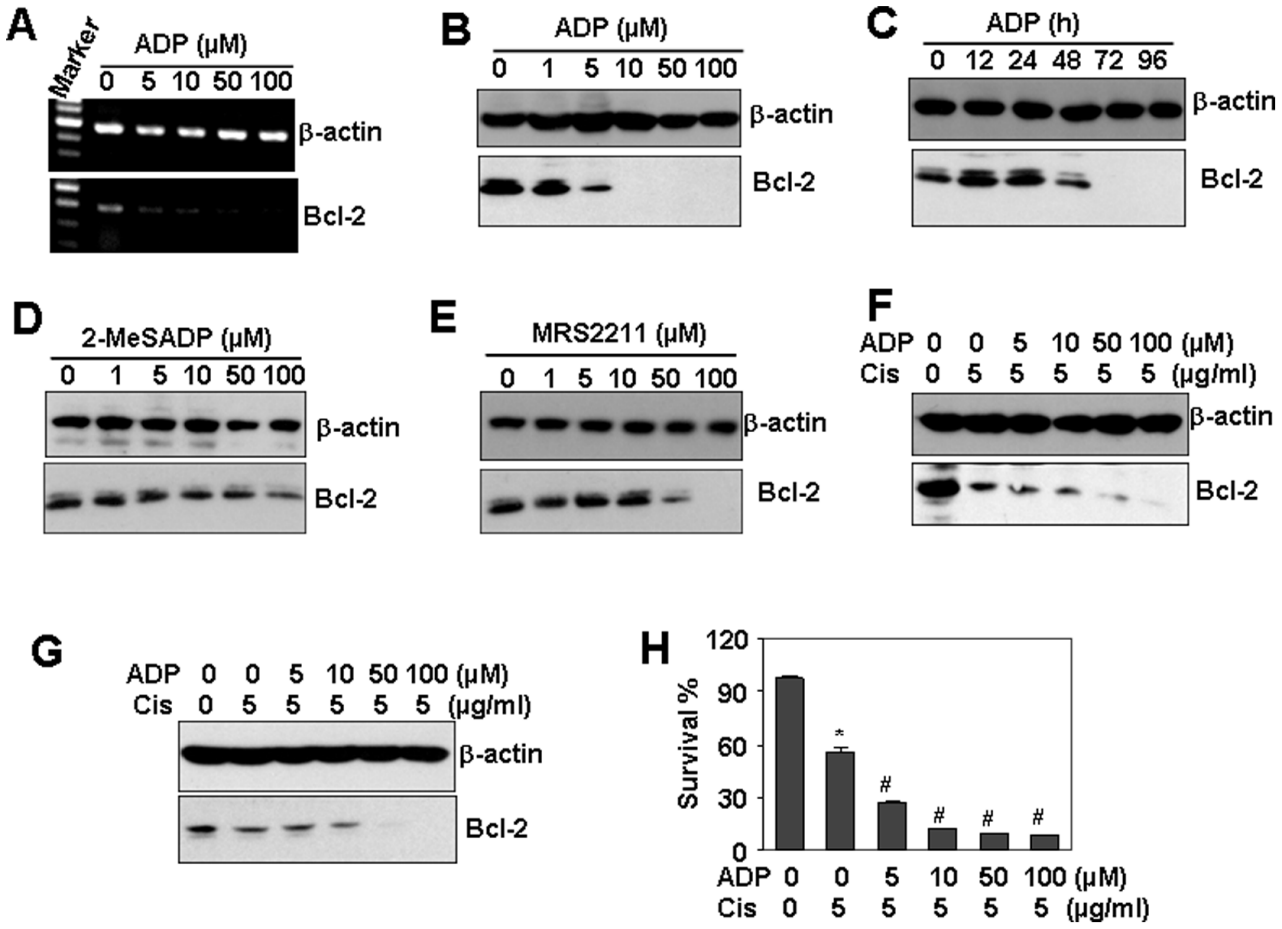
The effect of ADP on cisplatin-induced cell death. (A) ADP down-regulated the mRNA levels of Bcl-2. HUVEC were treated with indicated concentrations of ADP for 48 h. The mRNA levels of Bcl-2 was detected by RT-PCR. β-actin transcript was detected as loading control. (B, C) ADP down-regulated Bcl-2 protein expression in a dose- (B) and time-dependent (C) manner. (D) The effect of 2-MeSADP on Bcl-2 protein expression. (E) The effect of MRS2211 on Bcl-2 protein expression. (F) The effect of ADP pre-treatment on cisplatin-induced Bcl-2 down-regulation in HUVEC. Cells, pre-treated with the indicated concentrations of ADP for 48 h. were re-treated with 5 µM cisplatin for 48 h. Bcl-2 protein expression was detected by western blot. β-actin protein was detected as a loading control. (G) The effect of ADP pre-treatment on cisplatin-induced Bcl-2 protein expression in HAEC. (H) The effect of ADP pre-treatment on cisplatin-induced cell death. HUVEC were treated as same as (F). The cell survival was detected by CCK-8 assay. * *P*<0.05 compared with the control group. # *P*<0.05 compared with cisplatin-treated alone group.

Nucleolin is a binding protein involved in Bcl-2 stabilization [16], [50]. The down-regulation of nucleolin destabilized Bcl-2 mRNA and sensitized cells to apoptosis [51]–[52]. The observation that ADP down-regulated nucleolin protein level prompted us to propose that ADP may impact the expression of Bcl-2. This hypothesis was confirmed by the RT-PCR results which showed that ADP dose-dependently down-regulated the transcript of Bcl-2 (Figure 7A). ADP also dose and time-dependently down-regulated the protein level of Bcl-2 in HUVEC (Figures 7B–C). We also found that 2-MeSADP did not affect Bcl-2 protein level (Figure 7D), and MRS2211 down-regulated Bcl-2 protein level (Figure 7E). Moreover, the pre-treatment with ADP promoted Bcl-2 down-regulation induced by cisplatin, a strong and widely used cancer chemotherapy drug, in both HUVEC (Figure 7F) and THP1 (Figure 7G). Moreover, ADP pre-treatment promoted cisplatin-induced cell death in HUVEC (Figure 7H).

### The effect of ADP on HUVEC cell migration

**Figure 8:**
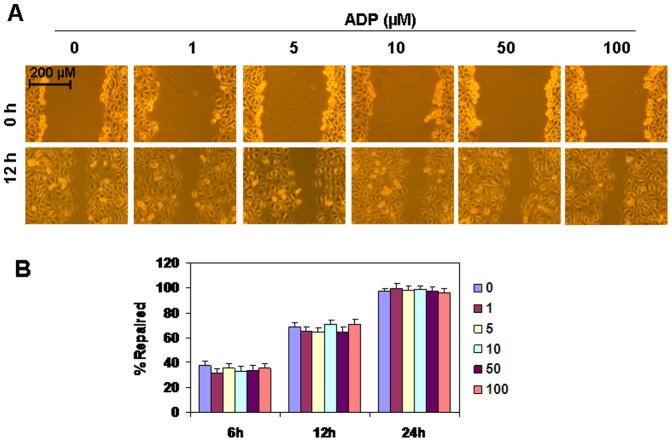
The effect of ADP on HUVEC cell migration. (A) HUVEC cells, grown to 100% confluence, were scratched to create a wound and then washed with medium to remove detached cells. Cells were then treated with the indicated concentration of ADP. The wound repairs in representative fields were photographed at time points 0, 12 h to assess the degrees of wound healing. Experiments were performed in triplicate and representative results were shown. (B) The quantitative repair data at time point 6, 12, 24 h.

ADP has been reported to regulate cell migration [53]–[54] via P2Y receptors. In this study, the results from scratch wound-healing assay showed that the treatment of HUVEC with 1–100 µM ADP for 24 h did not regulate wound healing (Figure 8A). Quantitative analysis data showed that repair percentages in ADP-treated groups were not significantly different from that in control groups at time point 6, 12, 24 h (Figure 8B). These results suggested that ADP did not regulate HUVEC migration.

### The effect of ADP on proliferation of cervical cancer cells

**Figure 9:**
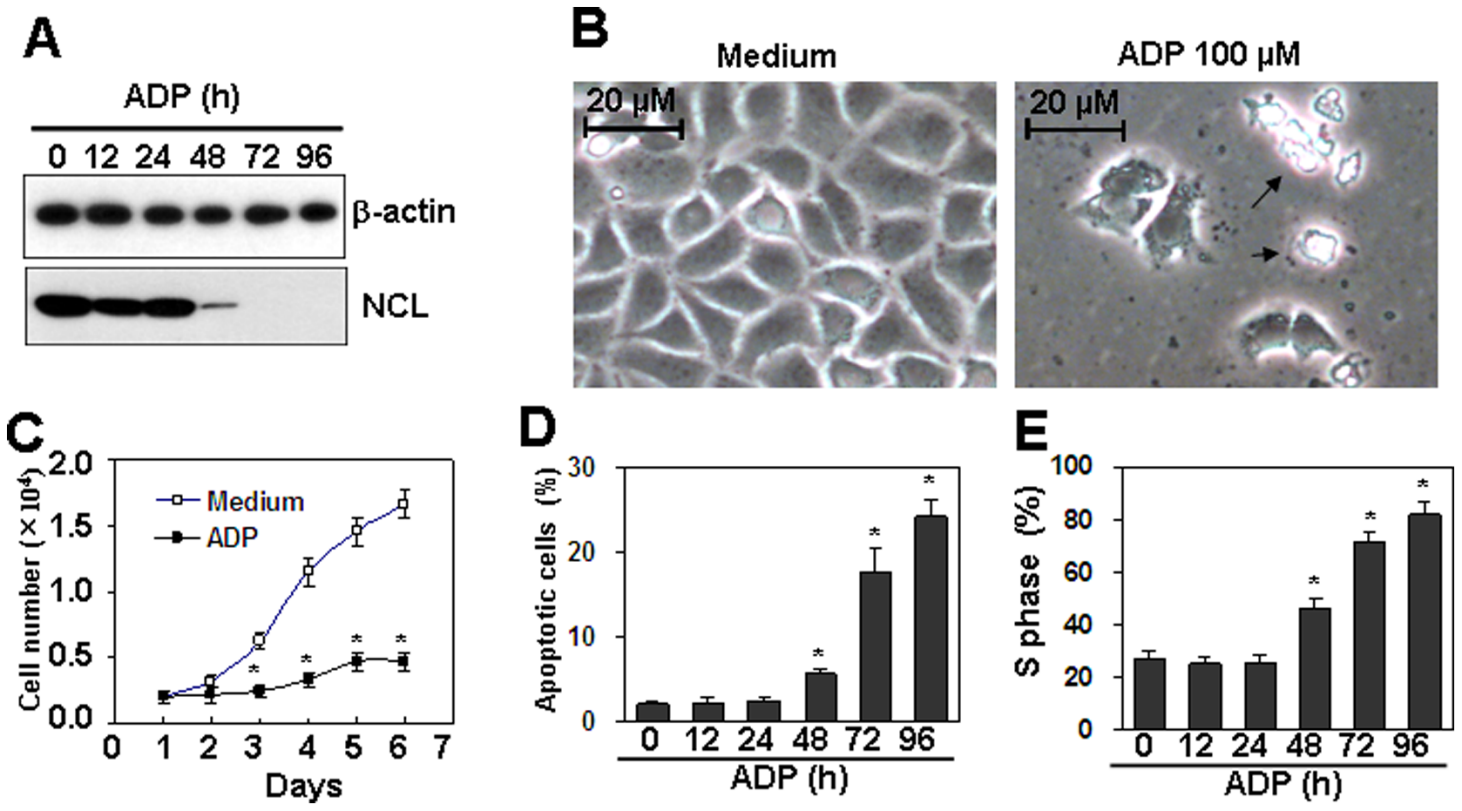
The effect of ADP on the proliferation of cervical cancer cells. (A) ADP down-regulated the protein levels of nucleolin in Caski cervical cancer cells. Cells were treated with 100 µM ADP for the indicated time periods. Nucleolin protein levels were detected by western blot. β-actin protein levels were detected as loading controls. (B) Microscope observation of Caski cells treated with ADP. Cells were treated with 100 µM ADP for 72 h. Cell morphology in the presented field was obtained by microscope. Arrows indicated the detached cell debris. (C) ADP inhibited proliferation of cervical cancer cells. Caski cells were treated with 100 µM ADP for the indicated time periods. Cell numbers were detected every day by CCK-8 assay. *P*<0.05 compared with the control group. (D) ADP induced cell apoptosis in cervical cancer cells. Caski cells were treated with 100 µM ADP for the indicated time periods. Cell apoptosis was measured by PI/FITC-Annexin V staining assay. *P*<0.05 compared with the control group. (E) ADP induced cell cycle arrest in S phase. Caski cells were treated with 100 µM ADP for the indicated time periods. Cell cycle was measured by PI staining. *P*<0.05 compared with the control group.

We found that ADP inhibited cell proliferation in HUVEC cells via induction of cell cycle arrest and cell apoptosis. As cell cycle arrest and apoptosis are benefit to cancer therapy, we tested the effect of ADP on cell proliferation of Caski cervical cancer cells. Western blot results showed that ADP dose-dependently down-regulated nucleolin protein levels in Caski cells as same as in HUVEC cells (Figure 9A). Cell number down-regulation and cell detached were observed by ADP treatment (Figure 9B). CCK-8 assay results showed that ADP treatment induced inhibition of cell proliferation in Caski cells as the same as in HUVEC cells (Figure 9C). ADP treatment also induced cell apoptosis and cell cycle arrest in S phase (Figure 9D–E). These results suggested that ADP may be valuable for cancer therapy.

## Discussion

In this study, we found that ADP down-regulated nucleolin protein, but not mRNA, in a dose- and time-dependent manner, suggesting that ADP induced a post-transcripional down-regulation of nucleolin. The nucleolin protein down-regulation was independent on P2Y1, 12, and 13 receptors. Nucleolin protein down-regulation was involved in ADP-induced cell arrest in S phase, cell apoptosis, and cell proliferation inhibition. Nucleolin protein down-regulation was also involved in the decrease of Bcl-2 expression, which sensitized cells to cisplatin-induced cell death.

Aptamers are short sequences of DNA or RNA that can bind to specific proteins via recognition of their three-dimensional structure. G-rich oligonucleotides (GROs), which function as nucleolin-binding aptamers, have strong growth-inhibitory activity against various types of cells by inducing S phase cell cycle arrest [44]–[45]. It has also been reported that GROs induce cell apoptosis in OE19 esophageal tumor cells [55]. In our study, we found that ADP down-regulated the nucleolin protein levels, which prompted us to suppose that ADP may induce cell arrest and cell apoptosis. As expected, ADP induced cell cycle arrest in S phase and cell death as GROs did. The over-expression of nucleolin by gene transfection in HUVEC partly, but significantly ameliorated cell arrest in S phase and cell apoptosis. We also found that ADP did not down-regulate the transcript of nucleolin, but down-regulated nucleolin protein from both the naïve and the transfected levels, suggesting that ADP might induce nucleolin degradation as aptamers did.

Nucleolin is a multifunctional protein that interacts with both DNA and RNA. By binding of its RNA binding and C-terminal domains to pre-rRNA, nucleolin functions as an assembly factor by bringing together the correctly folded rRNA and other components necessary for rRNA maturation and ribosome assembly [1]. It has been reported that nucleolin plays a critical role in mRNA stabilization. Nucleolin functions to stabilize Bcl-2 mRNA by protecting it from RNase degradation by binding to an AU-rich element (ARE) in the 3′-UTR of Bcl-2 mRNA in HL-60 [50] and chronic lymphocytic leukemia cells [56]. As Bcl-2 is an important molecule to regulate cisplatin-induced cell death, these studies provided insights into possible role of ADP to regulate cisplatin-induced cell apoptosis. In our study, we found that ADP down-regulated Bcl-2 expression at both gene and protein levels, and promoted HUVEC sensitive to cisplatin-induced cell apoptosis, suggesting that ADP may be an accessory agent for cisplatin chemotherapy.

Several studies have shown conflicting results about the effect of ADP on cell proliferation. ADP has been reported to promote vasa vasorum endothelial cell growth by induction of intracellular Ca^2+^ response and activation ERK, Akt and S6 ribosomal protein via P2Y1 and P2Y13 receptors [30]. ADP also has a neuroprotective function in the repair of retinal tissue following injury by enhancing cell division and inhibiting cell death via P2Y1 receptor [26]. However, ADP has been reported to inhibit cell proliferation in LXF-289 cells, an adenocarcinoma-derived cell line from human lung bronchial tumor, by activation of multiple mitogen-activated protein kinase pathways and nuclear factor kappa B1, and by arresting the cells in the S phase via P2Y receptors [28]. In this study, we found that ADP impaired cell proliferation in endothelial cells. The inhibition of cell proliferation was due to the induction of cell cycle arrest in S phase, and the induction of cell apoptosis.

Cell apoptosis and cell cycle arrest are two important mechanisms to inhibit cell proliferation. Extracellular nucleotides, including ATP, UTP, ADP and UDP, inhibit growth of oesophageal cancer cells via the induction of cell apoptosis and cell cycle arrest [57]. Adenine nucleotides, including ATP and ADP, have been reported to inhibit proliferation of the human lung adenocarcinoma cells via the induction of massive accumulation of cells in the S phase [28]. By acting at human P2Y1 receptor, adenosine nucleotides stimulate mitogen-activated protein kinases and induce apoptosis [31]. In pancreatic beta cells, the activation of ADP receptor P2Y13 induces cell apoptosis which can be reversed by the P2Y13 receptor specific antagonist MRS2211 [32]. Unexpectedly, ADP induces endothelial cell apoptosis via the activation of transcription factor NF-κB [34]. In our study, we found that ADP inhibited cell proliferation via the induction of apoptosis and cell cycle arrest in S phase. But this effect was not due to the activation of ADP receptors, including P2Y1, 12, and 13 receptors. First, HUVEC did not express P2Y1 and 12 receptors. Second, P2Y13 receptor inhibitor MRS2211, did not ameliorate ADP-induced inhibition of cell proliferation. On the contrary, MRS2211 itself induced cell proliferation inhibition as ADP did. Moreover, the inhibition of cell proliferation induced by ADP was related to the down-regulation of nucleolin. The over-expression of nucleolin reversed ADP-induced inhibition of cell proliferation. All three P2Y receptor inhibitors, including P2Y1 receptor inhibitor MRS2179, P2Y12 receptor inhibitor PSB0739, and P2Y13 receptor inhibitor MRS2211 did not reverse ADP-induced down-regulation of nucleolin, suggesting that P2Y1, 12, and 13 receptors were not involved in ADP-induced down-regulation of nucleolin. Furthermore, a P2Y1, 12, 13 receptor agonist, 2-MeSADP did not induce inhibition of cell proliferation as ADP did. All theses results suggested that ADP-induced inhibition of cell proliferation was not due to the activation of P2Y1, 12, and 13 receptors.

ADP has been reported to regulate cell migration [53]–[54] via P2Y1 or P2Y12 receptors. In this study, the results from scratch wound-healing assay showed that ADP treatment did not regulated HUVEC migration, which may be due to the non-expression of P2Y1 and P2Y12 receptors.

ADP has also been found to down-regulate cell proliferation in Caski cervical cancer cells by induction of cell cycle arrest in S phase and cell apoptosis, suggesting that ADP may be valuable for cancer therapy. However, ADP promotes blood coagulation by induction of platelet aggregation via activation of P2Y1, and P2Y12 receptors [18], further in vivo experiments will be needed for evaluating its in vivo effect for cancer growth.

In our study, we found that ADP treatment down-regulated the level of nucleolin protein, but did not down-regulate the expression of nucleolin transcript. We also found that ADP down-regulated the over-expressed nucleolin. These results suggested that ADP down-regulated nucleolin protein via post-transcriptional manner, which may also be the reason why nucleolin over-expression can not partly reverse ADP-induced cell cycle arrest, cell apoptosis, and cell proliferation inhibition.
